# A Case of Hemiplegia, Succeeded by Mania

**Published:** 1781-09

**Authors:** 


### SECTION II.

### ESSAYS AND OBSERVATIONS.

I. *A case of hemiplegia, succeeded by mania. Communicated by* Richard Cowling, *M. D. surgeon to the* 85*th regiment of foot. Read* August 27*th*, 1781.

WIlliam Jackson, a private soldier, twenty-two years old, was suddenly seized, on the 2d of Sept. 1780, with a great numbness on his left side, and immediately after perceived he had in a great measure lost the use of all his limbs of that side. He was carried to the hospital, where I found him the next morning labouring under a considerable stupor, and deprived of almost all voluntary power of the whole left side. He had however now and then slight involuntary twitchings, but no tremors, in both the upper and lower left extremities. In moving him from place to place he dragged his left leg behind him; and his left arm, which he was unable to raise in the smallest degree, was obviously much colder and paler, and seemed at the same time, time, though it had been difeafed only fo fhort a time, fomewhat fmaller than his right arm. The pulfe in the left arm was alfo much flower, fmaller, and feebler than that in the right. His tongue, and the parts about the larynx, were fo much affected as to occafion a faltering utterance in his fpeech, and a difficulty in fwallowing even his faliva. The left cheek and the left fide of his mouth were generally fpafmodically contracted toward his left fide; but fometimes his whole face was thrown, convulfively, into feveral directions and fome fingularly ludicrous diftortions, infomuch that even a faturnine fpectator found difficulty in maintaining his gravity. To be laughed at, or even fmiled at, feemed, from the louring look with which every obferver was conftantly eyed, what the patient moft dreaded, and what never failed, however triflingly and inadvertently excited, to put him into the moft immediate and outrageous paffion; while the fecretly retrograde manner, in which he always attempted to withdraw himfelf from every beholder, intimated that he was confcious, or rather, in the prefent ftate of his brain, inftinctively aware of his being an object of derifion. His appetite was moderate, his belly rather bound, but his nights were entirely fleeplefs.

His

His mind was equally affected with his body, fo that to the fimpleft and plaineft queftions he returned the moft incoherent anfwers. Every motion that he made with the found fide of his body was of a very vehement and defultory kind. His eyes wide open, and, feemingly protruding, glared a wild fierce ftare, and he was inceffantly grating his teeth with all the gefticulations of rage. Although he had loft the ufe of one fide, his conduct, notwithftanding, became that night fo violent as to render it prudent to appoint him two attendants, in preference to the ftrait waiftcoat, which, from the different applications that might be neceffarily made to his left fide, was deemed inconvenient. The lifelefs immobility of his left fide, contrafted with the appearances juft now defcribed, formed a fight as fingular as it was melancholy.

Upon inquiring of his comrades it did not appear, that he had ever, to their knowledge, been affected with any indifpofition fimilar to this, but they all agreed, that he had been guilty of frequent and violent intoxication, fpending the money he had got by making fhoes for the company (generally a guinea a week) and likewife his half week's pay in liquor, between Saturday and Monday; and that he had been known, after thefe dedebauches, to ſtarve for twenty-four and in ſome inſtances even forty-eight hours. Theſe circumſtances and the whole of the caſe conſidered, I had no doubt that this patient laboured under a complete hemiplegia, and what Dr. Battie would term, as being ſubſequent to the hemiplegia, a *conſequential* madneſs. And from the extremely thin habit of the patient's body, the well-proportioned ſize and length of his head and neck, his immoderate and repeated intoxications, and the ſtate of inanition that had ſucceeded them, I was of opinion, that his paralyſis was not to be attributed to a ſanguineous or ſerous compreſſion on the brain, but to a collapſe * of a part of the brain and nervous ſyſtem, brought on by the above-mentioned ſedative and debilitating cauſes: for it is a well known, though ſingular fact, both from the diſſections of paralytic ſubjects and the experiments made on brute animals, that one half of the brain and the oppoſite half of the body have been exceedingly injured, while the other half of the brain and of the body have not been at all affected.

Being convinced therefore, that my attention ought to be directed ſolely to the removal of

* *Collapſe* may be defined, a morbid diminution of the tone of the brain and of the motion of the nervous fluid.

 the the primary diſeaſe, I directed a bliſter to be applied to the patient's left arm and another to his left thigh, and at the ſame time ordered his whole left ſide to be well rubbed with a fleſh bruſh, and then wrapped up in flannel. Once in four hours he took a bolus compoſed of gum guaiac. gr. v. limat. ferri gr. xv. and pulv. cort. Peruv. ʒſs. and he waſhed down each bolus with four table ſpoonfuls of the following formula—℞. Tinct. canthar. ℥j. ſal. c. c. vol. ℥ſs. tinctur. aromat. ℥ij. aq. menth. pip. ſimp. ℔jſs. M.——For his breakfaſt he was allowed a pint of milk, five ounces of bread, and rather a large proportion of nutmeg; for dinner, a pound of fleſh-meat, ſix ounces of bread, and a pint of porter, with a large quantity of pepper, muſtard, and horſe-radiſh; and for ſupper, he had, repeated, his breakfaſt. He went on with all theſe meaſures for four days, without experiencing one lucid interval, or any other alteration, than, on the laſt of thoſe days and the ſixth of his diſeaſe, a tumefaction of his whole tongue, and an almoſt unintelligible muttering in his ſpeech: his tongue, which had acquired a morbid inclination toward his left cheek, and which had likewiſe loſt its uſual volubility and ſenſation, had been bit through in ſeveral places by the grinding of his teeth, withwithout the patient having even once made the least complaint. These wounds in his tongue were washed with a solution of alum and nitre.

As I had met with very great success, in four former but pure palsies, from this patient's regimen and medicines, I determined to persevere steadily in their use; and, to give them greater effect, now further ordered his left side and left limbs to be frequently rubbed with a mixture of vinegar and mustard.——In this manner he was treated during the remainder of his disorder. At length, on the eleventh day, it was observed, that, along with other signs of a general amendment, the pulse, in particular, in the patient's palsied arm, was become stronger and fuller, and the muscles of the same side firmer and more elastic than they had ever been before since his attack, and on the day following his reason, which had been hitherto so exceedingly disordered, appeared to be proportionably restored; the grinding of his teeth had ceased, the whole of his conduct was more calm and consistent, and his speech seemed to be much mended.

From the time that the above salutary change took place, the patient continued gradually, though slowly, to recover; first, the use of his limbs, and, soon afterwards, the faculties of his

 mind, mind, so that on the twenty-first day from the commencement of his disorder he was discharged, cured.

*Furnival's Inn,*
July 13th, 1781.

